# Primer on disability: Why accessibility is important for all medical physicists

**DOI:** 10.1002/acm2.70003

**Published:** 2025-02-09

**Authors:** Lindsay E. Jones, Grace Eliason, Shivani Gupta, Elizabeth G. Jeong, Abigail Besemer, David A. Sterling, Jessica M. Fagerstrom

**Affiliations:** ^1^ MedPhys Lindsay, LLC Baltimore USA; ^2^ Department of Radiology University of Colorado School of Medicine Aurora USA; ^3^ Medical and Radiation Physics, Inc. San Antonio USA; ^4^ Department of Radiation Oncology Washington University School of Medicine St Louis USA; ^5^ Department of Human Oncology University of Wisconsin Madison USA; ^6^ Department of Radiation Oncology University of Minnesota Minneapolis USA; ^7^ Department of Radiation Oncology University of Washington Seattle USA

**Keywords:** accessibility, accommodations, disability, leadership, professionalism

## Abstract

Disability and accessibility remain under‐addressed topics despite the increasing prevalence of disabled people in the workforce. In the field of medical physics, there is growing evidence that the proportion of people with one or more disabilities mirrors that of the US population. Addressing disability and accessibility is a crucial facet of the American Association of Physicists in Medicine's 2018 strategic goal to “champion equity, diversity, and inclusion in the field of medical physics.” This review aims to provide guidance on disability‐related topics in the context of the medical physics profession. An overview of current knowledge and recommendations is provided on essential topics such as how to comply with federal law, handle accommodation requests, and discuss disability using appropriate language. To that end, background information such as definitions, models, and classifications of disability is included. Beyond the essentials, this review also applies disability‐related concepts to improve overall efficiency and productivity, attract diverse talent, and demonstrate leadership as an individual or organization.

## 1 INTRODUCTION

Disability and accessibility within the profession of medical physics have yet to be comprehensively reviewed in existing literature. This gap in knowledge reflects a broader trend in which the specific experiences and needs of disabled professionals are overlooked.^1^, ^2^, ^3^ As a result, there is a lack of data and understanding that could inform more inclusive practices and policies within the discipline. Addressing this oversight is essential to fostering a more equitable and supportive environment for all physicists.

In line with AAPM's 2018 focus area of diversity and inclusion, with the strategic goal of championing equity, diversity, and inclusion in the field of medical physics,^4^ the Accessibility Subcommittee was established under the Equity, Diversity, and Inclusion Committee (EDIC) of the AAPM in 2022. AAPM's support of the EDIC and its subcommittees signifies a commitment to improving accessibility and inclusion within the profession. The subcommittee aims to identify barriers faced by disabled medical physicists and to develop strategies for mitigating these obstacles. This proactive approach highlights the importance of creating an environment in which all individual physicists can contribute and thrive.

It is pivotal that all professionals recognize the significance of disability and accessibility in the medical physics field. Disability is one of the few marginalized groups that anyone can become part of at any stage of life, and most people will experience it at some point. Statistics indicate that more than 25% of adults in the US have one or more disabilities,^5^ and the medical physics community mirrors these demographics.^6^ As such, medical physicists should work toward a transition from mere awareness of disability to a commitment to creating accessible and inclusive professional communities.

This review article is intended to serve as an introductory guide for both disabled and non‐disabled medical physicists. It overviews key concepts related to disability, including appropriate language and terminology, as well as practical recommendations for accommodations in professional medical physics settings. By enhancing understanding and fostering an inclusive culture, this guide seeks to ensure the field of medical physics benefits from the diverse talents and perspectives of all its members. Through these efforts, a more inclusive and supportive professional community can be achieved.

### 1.1 Models and definitions of disability

**FIGURE 1:**
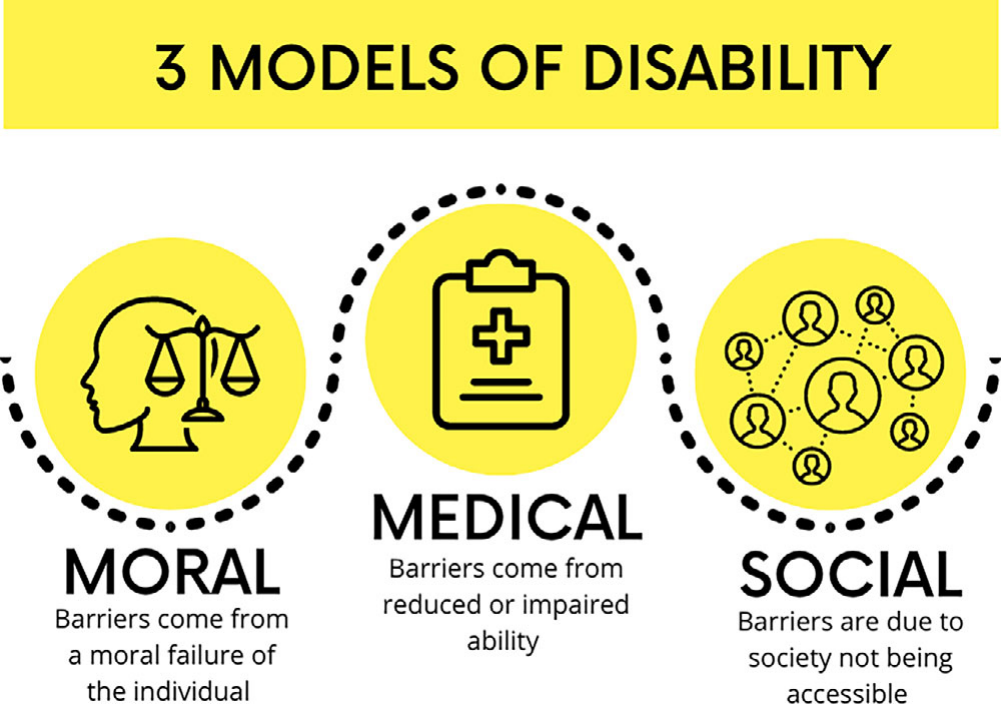
The moral, medical, and social models of disability.

There are currently three major models through which disability is viewed: the moral model, the medical model, and the social model (Figure 1).^7^

The moral model posits that disability results from a moral failure of some kind, the specifics of which vary based on society's values.^7^ Traditionally tied to a belief system, this model attributes disability as a punishment for sin or other wrongdoing by a disabled individual or their family.^8^ Through a secular, contemporary lens, the moral model views disability as a failure of willpower, tragedy or inspiration for non‐disabled people, or the result of not achieving wellness ideals. The latter examples are commonly accepted and can be quite harmful, acting as justification for depriving individuals of healthcare, reasonable accommodations, and bodily autonomy. Thus, regardless of the moral framework used to judge, the moral model fundamentally conceptualizes disability as the result of individual moral choices, rather than a solvable failure of society at large.

Similarly, the medical model conceptualizes that the barriers a disabled person faces are due to their reduced or impaired ability.^7^ It holds that disability results from an individual person's physical or mental limitations, unconnected to their environment and society. For example, a medical physicist with arthritis may struggle lifting equipment because they cannot utilize the handle, in which case, the disabled person is expected to adjust their approach to the intended design of the equipment. Despite seeming more objective and sensitive than the moral model, the medical model places the onus on the disabled person's deficits rather than the design of the world around them.

The Centers for Disease Control and World Health Organization define disability as any condition of the body or mind that makes it more difficult to do certain activities and interact with the world.^9^ Similarly, the Americans with Disabilities Act (ADA) defines a person with a disability as someone who: has a physical or mental impairment that substantially limits one or more major life activities, or has a history or record of an impairment, or is regarded as having such an impairment by others.^10^, ^11^ Both are examples of the medical model of disability and are primarily meant to serve as civil rights protections under the law.

The currently accepted model of disability within the disability community is the social model, which says that barriers disabled people face are because society is not designed to be accessible.^7^ Where viewing disability through the medical model would focus efforts on changing the disabled person, the social model of disability focuses efforts on reducing societal barriers. For example, requiring eyeglasses to correct vision could be considered a disability under the medical model, but the widespread availability of affordable eyeglasses in the US has largely removed societal barriers for individuals with mild visual impairments, normalizing and making it rarely perceived as a disability. In the previous example in which a disabled medical physicist struggles to lift equipment using a handle, the social model views the design of the device as the barrier to access as opposed to the disabled person's abilities. Addressing this situation through the social model lens, the principle of universal design^12^ could be employed to develop a more accessible handle for all users, reducing occupational accidents and improving efficiency for all. Many disabled people prefer the social model of disability, or a strengths‐forward, non‐deficit model. Using the social model removes stigma from disability and focuses efforts on addressing societal barriers to access and equity for disabled people.

These models of disability have coexisted throughout history, with evidence of care for disabled individuals in Native American communities and Colonial America.^13^ The development of modern science in the 18th century and its application to evidence‐based medicine revolutionized popular views of disability, shifting perspectives from the moral toward the medical model. The medical model brought many material benefits–treatment, assistive devices, and education–as well as reducing the social stigma associated with the moral model.^14^ However, the medical model also became associated with eugenics, which sought to eliminate perceived defective traits in the population through methods such as forced sterilization.^15^ In the 20th century, the scientific community rejected eugenics in favor of ethical perspectives based around individual dignity and autonomy. Along with movements for equality based on race and gender, disabled people have advocated for inclusion in society by reframing disability with the inclusive social model the community favors today.^16^ Many accessibility solutions like braille on signs, curb cuts on sidewalks, and ramps to enter stores and institutions only became common after the ADA mandated accessibility of public spaces.^17^ This brief historical overview highlights the evolution of some disability models, illustrating how societal views and interactions with disabled individuals have changed over time and how remnants of earlier perspectives continue to present challenges today.

### 1.2 Classification of disability

**FIGURE 2:**
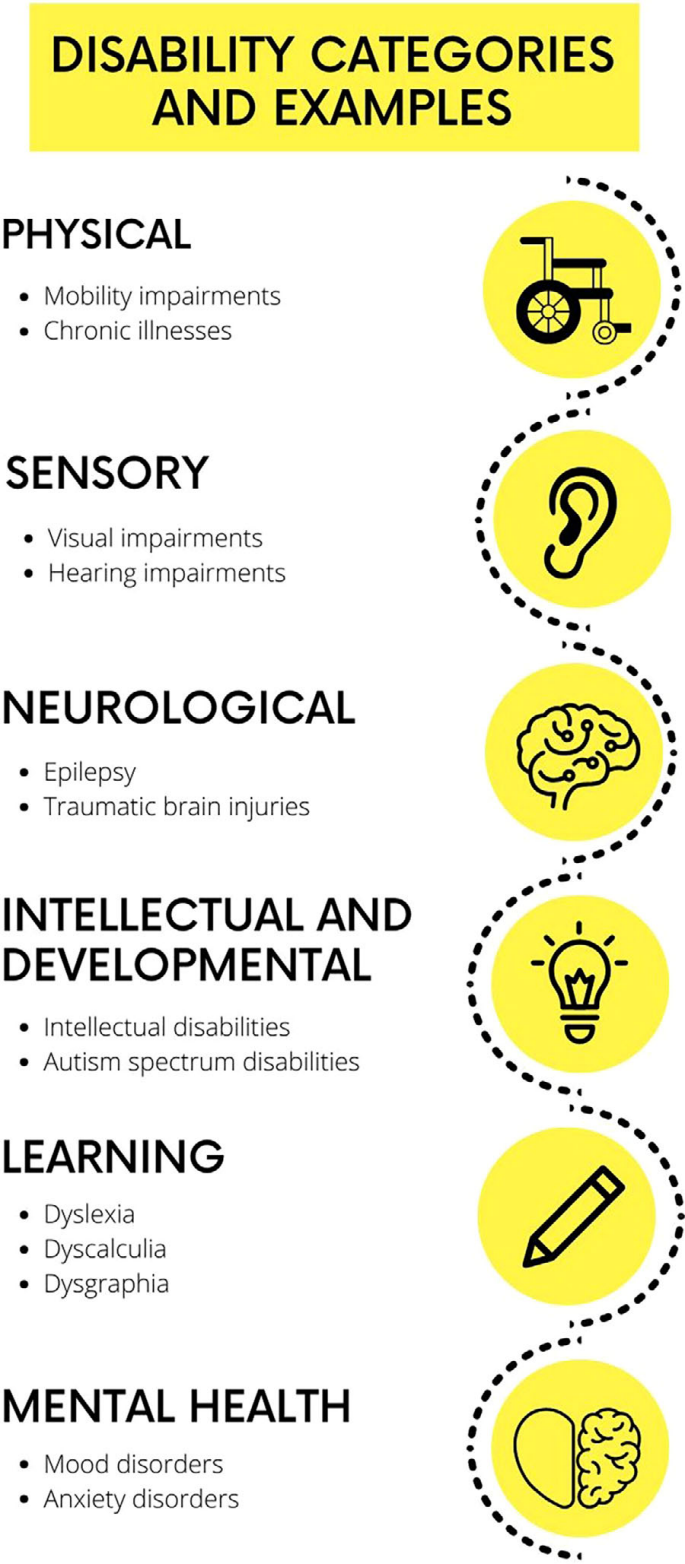
Disability categorized by manifestation with examples.

No singular categorization scheme for disability has been widely accepted. Various groupings have been made based upon support needs as well as medical descriptors, paralleling the social and medical models of disability, respectively. For instance, the CDC characterizes demographic patterns in disabilities with categories of cognition, hearing, mobility, vision, self‐care, independent living, and other/any.^5^ These categories differ from those used in education, such as the thirteen defined by the Individuals with Disability Education Act.^18^ To provide a comprehensive guide, we have broadly grouped disability into six categories based upon manifestation: physical, sensory, neurological, intellectual and developmental, learning, and mental health (Figure 2). A more complete list of disabilities as well as accommodations can be found on the Job Accommodation Network website.^19^

Disability can also be described by apparentness to others. Disabilities that cannot be instantly seen–or are less readily apparent to others–are referred to as invisible or non‐apparent. Non‐apparent disabilities are quite common. In 2020, of the 14 million UK adults who reported having a disability, 70% listed theirs as non‐visible.^20^ A disability may also be described by its duration of effects, which can be permanent, temporary, or situational. For example, auditory impairment could be experienced permanently due to deafness, temporarily due to an ear infection, or situationally in a loud environment. Disability can also be described as static, such as a congenital absence of limbs or limb amputation, or dynamic, such as autoimmune or other chronic illnesses that affect fatigue or pain at varying levels over time.

Lastly, terms such as “severity” and “functionality” used to describe disability have been replaced by a support needs‐based model.^21^ These terms are still deployed under the medical model of disability, but they have many limitations, including lack of flexibility, an overemphasis on individual impairments, subjectivity in interpretation, and the potential to reinforce negative stigma by oversimplifying the complex, multidimensional nature of disability and ignoring broader social and environmental factors. The support needs‐based model describes the type and level of accommodations an individual requires to effectively engage with their surroundings. This holistic approach is also more fitting for those who have more than one disability, which accounts for 12% of the US adult population.^22^

Characterizing a disability can be complicated. Take, for example, postural orthostatic tachycardia syndrome (POTS), a type of autonomic nervous system dysfunction (dysautonomia) that causes an abnormally rapid increase in heart rate upon sitting or standing.^23^ Dysautonomias can cause symptoms like neuropathic pain, fatigue, racing heart rate, excess sweating, weakness, dizziness, and fainting episodes. In a medical physics workplace environment, these symptoms might be difficult to perceive, and this syndrome could be classified as a non‐apparent disability. The duration of POTS is assumed to be life‐long because it currently has no known cure. It can be a dynamic disability, where the symptoms fluctuate daily or hourly depending on many factors: ambient temperature, hydration and electrolyte status, frequency of meals, wearing of compression garments, the amount of time spent sitting or standing, and the effects of medications. Support needs can vary widely—some people with POTS can manage their condition with hydration and compression garments, some need medications, and some require accommodations to their work environment. Such accommodations could include allowing them to lay down horizontally, work in a reclined workstation, and/or work from home. Someone with POTS may have fainting episodes and require a service dog to alert them of imminent syncope. The same diagnosis can impact individuals in different ways. As a result, even when two physicists have the same diagnosis, the support and accommodations that they require may differ significantly.

### 1.3 Ableism

Ableism, defined as discrimination and social prejudice against individuals with disabilities, is predicated on the belief that individuals with typical abilities are superior. This ideology is grounded in the assumption individuals with disabilities require fixing and are primarily defined by their disabilities.^24^ Similar to racism and sexism, ableism categorizes entire groups of people as inferior, which perpetuates harmful stereotypes, misconceptions, and generalizations. In medical physics, it is crucial to recognize and address ableism to foster an inclusive and equitable environment for all professionals and patients. Common manifestations of ableism include non‐compliance with disability rights laws, such as the ADA, and the failure to integrate accessibility into building design plans. Other forms of ableism encompass the assumption that people with disabilities desire or require fixing, mocking individuals with disabilities, and refusing to provide reasonable accommodations. Moreover, denying employment to qualified physicists who meet job requirements based solely on their disability constitutes a significant form of ableism, alongside illegal workplace discrimination.

Casual ableism can also manifest in everyday situations. Examples include selecting inaccessible venues for meetings or events thereby excluding some participants, using another person's mobility device as an armrest or footrest, and framing disability as either tragic or inspirational. Other instances involve occupying an accessible bathroom stall when a non‐accessible stall could be used without pain or risk of injury, wearing scented products in scent‐free environments, and communicating with individuals with disabilities in a patronizing manner. Additionally, asking invasive questions about medical history or personal life, assuming disabilities must be visible, and questioning the validity or extent of someone's disability are all examples of casual ableism. Disabled medical physicists face these questions in a variety of environments, including during interviews, work, and professional conferences. Each of these contributes to a culture of ableism.

Microaggressions are subtle, everyday verbal or behavioral expressions that convey negative slights or insults related to someone's gender, identity, race, sex, or disability. In the context of ableism, examples include phrases such as, “that's so lame,” “she is crazy,” or “you're acting so bipolar today.” Although these are often unintended, such comments and actions can significantly impact their recipients.^25^ Phrases such as “it's like the blind leading the blind” or “my ideas fell on deaf ears” imply that disability diminishes a person's value and that disability is inherently negative. Using words associated with disability as slurs or insults contributes to the stigma surrounding disability, reinforcing harmful stereotypes. As an example, calling a physicist “being OCD about the tank” for describing exacting requirements of equipment positioning prior to TG‐51 linear accelerator quality assurance not only minimizes the very real mental health condition of obsessive‐compulsive disorder but also dismisses important elements of patient safety. It is imperative to adopt language that respects and acknowledges disability as a natural and inevitable aspect of the human experience. A discussion of best practices for language use in the context of disability and accessibility within professional medical physics is presented in the following section.

### 1.4 Language

Effective communication is a key component of accessibility and inclusion. The language used to discuss and interact with individuals with disabilities shapes perceptions and attitudes. Thoughtful and respectful language choice is essential to fostering an inclusive environment where all individuals feel valued and understood. The choice of words and phrases can influence how disability is perceived and can either contribute to or diminish the stigma associated with it. By understanding and implementing best practices in language use, professionals can create a more inclusive and supportive environment for colleagues and patients with disabilities.

Language not only conveys information, but also implies judgment about a given topic, especially when a variety of terms are available. The language around disability has historically stigmatized disabled people and treated disability as a taboo subject, per the moral model of disability, underlying the importance of making conscious choices when speaking about this subject. As with many topics where attitudes are continually evolving, the language around disability changes often, with new terms arising or being reclaimed, while others acquire negative connotations. Rather than fixating on memorizing “correct” terms, what is most consequential is understanding how language can affect inclusiveness and approaching discussions around disability with respect and care.

In general, disability should be thought of as a natural part of the human condition, including statistically likely variations, akin to having freckles or being left‐handed. Therefore, when speaking about disability, it is best practice to choose terms that are neutral, straightforward, and devoid of judgment. For instance, the statement “Bob uses a wheelchair” is more neutral than “Bob is crippled.” When engaging with or discussing a person with a disability, it is important to avoid questions like “What's wrong with you?” Instead, if you must know the information for a particular reason, ask “What is your disability?”. This acknowledges that there is nothing inherently wrong with the person. Language should not be based on pity, remorse, or inspiration, as this can be demeaning and disrespectful. The National Center on Disability and Journalism provides a useful disability language style guide on their website to aid in using appropriate and respectful language.^26^

Disabled people, like any group, are not a monolith; they vary in individual opinions and preferences. Some prefer person‐first language, like “person with disabilities,” while others prefer identity‐first language, like “disabled person.”^27^ Academic works, especially those surrounding a disease or population, often employ person‐first language, while some disabled individuals and communities reclaim identity‐first language.^28^ Person‐first language is often chosen to reduce the dehumanization of disabled individuals, emphasizing the person over the disability. On the other hand, identity‐first language, preferred by some individuals, celebrates pride and identity by placing the disability at the forefront. These preferences for person‐first or identity‐first language can vary across individuals and different disability communities based on historical context, cultural influences, and the degree of empowerment associated with embracing one's disability identity.^28^ Whenever possible, it is advisable to ask how an individual prefers to be referred to. And lastly, when disability is not pertinent to the conversation, it is best left omitted entirely.

### 1.5 Disability in the workforce

There is a significant gap in workforce participation between disabled and non‐disabled people.^29^, ^30^ Disabled people may experience discrimination in hiring, firing, promotion, and other employment decisions. Disability discrimination was the third most common type of claim workers reported to the US Equal Employment Opportunity Commission in 2020, behind only retaliation and age.^31^ Part of this discrimination includes disabled Americans experiencing difficulty receiving reasonable accommodations in the workplace. All such issues are compounded for those with additional marginalized identities.^32^

According to the US Bureau of Labor Statistics,^2^ the 2023 unemployment rate for adults without a disability was 3.6%, while those with a disability experienced an unemployment rate of 7.2%. More specifically, disabled adults who identified as white experienced the lowest unemployment rate at 6.7%, compared to Hispanic disabled people at 9.2% and Black disabled people at 10.2%. Overall, disabled people are a growing share of the labor force, seeing a sharp increase with COVID‐19 starting in 2020.^2^, ^33^

Effective practices for fostering an inclusive environment involve thoughtful consideration and proactive communication. Most importantly, it should not be assumed that all individuals participating in a space are non‐disabled. When organizing medical physics conferences, business meetings, networking events, or professional social gatherings, the accessibility of the venue and event should be evaluated from the perspective of individuals with physical, sensory, and neurological disabilities. Colleagues should be asked proactively about their needs to ensure their comfort, allowing sufficient time for responses through written or oral means. Best practice is to provide accessibility information as well as contact information for questions regarding accessibility prior to an event or meeting.

### 1.6 Interview and meeting preparation

Effective preparation for interviews and meetings is crucial to ensuring accessibility and inclusivity within the field of professional medical physics. A comprehensive approach to accessibility considers not only physical modifications but also adjustments in communication methods and sensory environments. By recognizing and addressing the diverse needs of participants, organizations can foster a more inclusive atmosphere that supports full participation from all individuals, including those with disabilities.

The social model of disability can be employed to guide interactions with individuals requiring accommodations.^34^ Questions such as, “How can participation be facilitated for you?” or, “What can we provide to make you most comfortable?” are encouraged. Emphasis should be placed on proactively modifying the meeting environment or the delivery of information to meet the needs of disabled participants, thereby reducing the necessity for them to request accommodations. Proactive adjustments can include anticipating accessibility needs and incorporating them into planning stages, ensuring a more inclusive experience for all participants. By adopting this approach, barriers to participation are minimized, promoting a more welcoming atmosphere. The goal is to create an environment where the needs of disabled individuals are met seamlessly, reflecting a commitment to inclusivity and accessibility in professional settings.

Access barriers extend beyond physical obstacles to include programmatic accessibility, defined by an organization's policies and practices.^35^ To achieve programmatic accessibility, it is essential to eliminate barriers related to communication and sensory experiences.^36^ The ADA mandates that covered entities maintain effective communication with individuals with disabilities. Communication barriers can arise from various issues, such as missing or inaccurate captioning, use of technical jargon instead of plain language, websites incompatible with screen reading software, and lack sign language interpretation. Sensory barriers may also pose significant challenges, and include practices such as color‐coding, which is inaccessible to colorblind individuals, strobe lighting, which may trigger seizures, and failure to provide audio descriptions of visual content for those who are blind. Additionally, the use of fragranced cleaning or personal care products can contribute to sensory overload or cause severe reactions in some individuals, such as migraines or anaphylaxis. Creating accessible spaces and events can be achieved by applying universal design principles and involving individuals with disabilities in the planning process. Numerous online resources are available to guide the hosting of accessible events.^37^, ^38^, ^39^, ^40^

### 1.7 Legal considerations for employees and employers

Federal law protects individuals with disabilities from discrimination. Disclosure of a disability to an employer is not required during the job application process or upon hiring, even if a reasonable accommodation is later necessary. It is unlawful for employers to refuse to hire or promote, to terminate or demote, to harass, or to pay less to an individual based on their disability, provided the individual is capable of performing essential job functions with reasonable accommodations.^41^ Additionally, employees are protected from unnecessary medical inquiries in the workplace. Under these laws, individuals are entitled to request and receive reasonable accommodations, which ensures equal opportunity for access and success.

The US Equal Employment Opportunity Commission enforces federal laws barring discrimination against many characteristics, including race, religion, sex, national origin, age, and disability.^42^ They publish guidelines for how to comply with the aforementioned laws, including a list of prohibited policies and practices.^43^, ^44^ The ADA of 1990 was the landmark civil rights legislation for disabled people in the United States. It was amended in 2008 and named the ADA Amendments Act of 2008.^10^

Title 1 of the ADA prohibits discrimination against qualified applicants with disabilities. It applies to private employers with 15 or more employees, as well as to all state and local government employees. Strict confidentiality requirements are mandated by the ADA, necessitating that medical information disclosed during the hiring process, whether pre‐ or post‐offer, be kept confidential with specific exceptions. These requirements ensure the protection of both voluntarily revealed information and information disclosed in response to an employer's questions, whether written or oral, or during a medical examination.

The ADA does permit some exceptions to confidentiality. Employers may share medical information with decision‐makers involved in the hiring process who require it to make employment decisions in compliance with the ADA. Additionally, supervisors and managers may be informed of necessary work restrictions and reasonable accommodations, while first aid and safety personnel may be notified if the disability might necessitate emergency treatment. The ADA also allows the sharing of medical information with government officials investigating compliance, state workers' compensation offices, state second injury funds, or workers' compensation insurance carriers. Furthermore, employers may use the information for insurance purposes.^45^

### 1.8 Accommodations requests

Accommodations is a broad term that refers to any equipment, services, facilities, and resources that can help provide a disabled person with support they need to make their lives easier and help them navigate their environment. Accommodations requests will be handled differently depending on the organization, be it a clinical consulting firm, company in industry, or large academic institution. For example, the pathway may be overseen via an accessibility office, human resources department, or an individual supervisor. If you are in the position of recruiting candidates or hiring decisions, you should have a basic understanding of how accommodations requests should be submitted, so that you could advise anyone seeking that information. Accommodations might be requested for graduate school or residency interviews, classroom or working environments, or clinical testing conditions.^46^ In employment situations, there may be a need for physical accommodations, such as adaptation of quality assurance equipment, the need for additional ancillary equipment, or access to accessible parking. Requested accommodations can also be for sensory, neurological, or mental health disabilities, such as changes in office lighting, workplace acoustics, or the ability to request time off or work‐from‐home days without retaliation or consequences.^47^ An up‐to‐date list of accommodations can be found on the Job Accommodations Network website.^19^

Accommodation requests allow for creative and iterative discussion between the disabled person and their employer. Keep in mind that multiple people with the same diagnosis/diagnoses may not need the same accommodation(s) to succeed in their respective positions. It is also important to acknowledge that requiring very specific documentation from medical professionals for granting accommodations requests puts a (perhaps undue) burden on disabled people—oftentimes folks with non‐apparent and/or dynamic disabilities have difficulty getting physicians to believe them and document their issues. Consider having a set of “universal”, that is, no documentation necessary, basic accommodations available for your students/residents/employees. Some examples of this include additional time on exams or projects, permission to record meetings or lectures, and accessible workspaces.

One thing to consider when someone makes an accommodation request is if there is a way you can offer their proposed solution to everyone. This approach shifts from accommodation, which addresses individual needs, to access, which creates an inclusive environment for all. For example, if an employee has a need for regularly scheduled work‐from‐home days to manage fatigue, anxiety, or any other issue, perhaps this can be an opportunity to offer flexible scheduling to all staff members. This approach incentivizes universal design and creating systems, spaces, and expectations that benefit everyone.

### 1.9 How to be a strong leader with respect to disability?

Effective leaders recognize that diverse teams are strong teams,^48^ and disability is a facet of diversity. For leaders looking to create diverse and inclusive teams, it is crucial to understand that individuals living with disabilities know best what they need. It is not the leader's job to be an expert on ableism or diagnosis by judging the validity of requested supports or accommodations, but rather to listen, learn, follow best practices to support their mentees, trainees, and colleagues in securing the access they need to succeed. Those with strong leadership skills—initiative, adaptability, service orientation—are likely already fully capable to recruit and bolster disabled people on their team.

**FIGURE 3:**
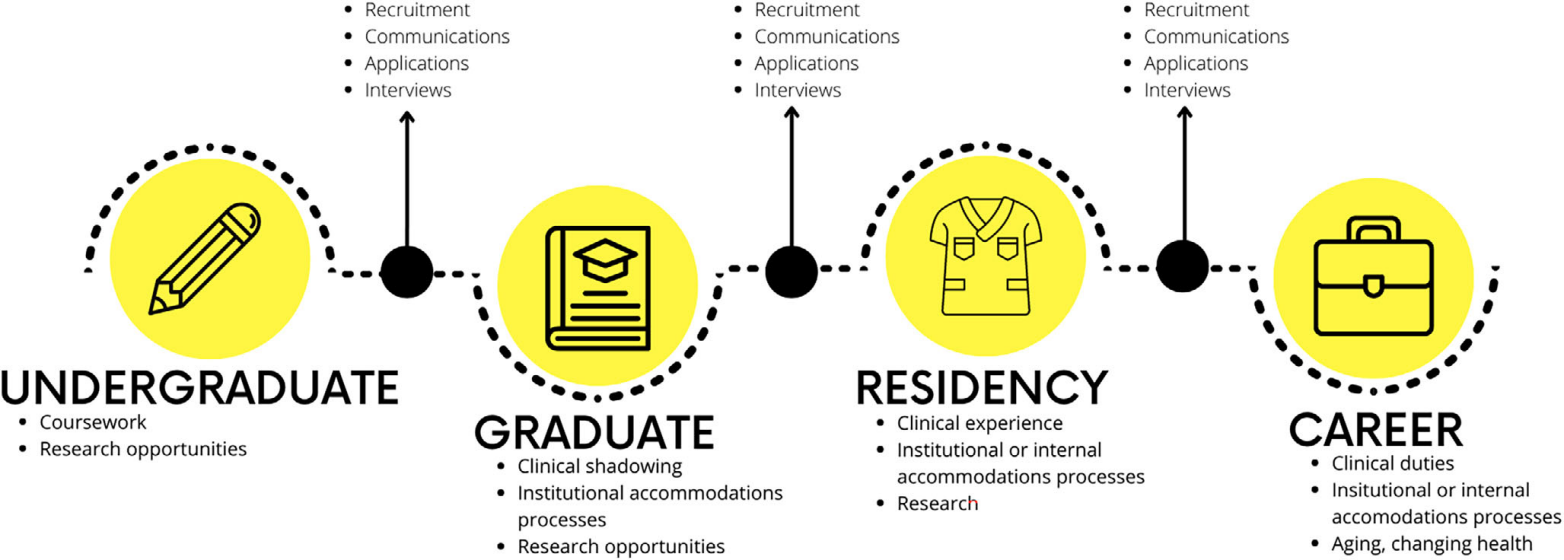
Opportunities for accessibility along the career of a medical physicist.

There are key points in the career cycle of a medical physicist wherein a leader can be in the position of championing a disabled candidate or can leave the candidate feeling unwelcome (Figure 3). Recruitment for graduate students, residents, and career positions are steps at which leaders can make a large difference if they foster an environment of inclusivity.^49^ One way to advertise this would be to disclose the locations of interviews and what the physical accessibility of those spaces is like, can be done directly in the job listing. The same listing could include the contact information for whomever would ensure access and/or accommodations for an interview. These actions signal to potential candidates that you are taking accessibility seriously.

Being proactive and considering disabled trainees or employees in your planning process and making the environment comfortable for people to approach you with questions are good first steps to establish yourself as a leader with respect to disability and diversity.

### 1.10 Key points and actionable recommendations

- There have been many historical lenses through which disability and accessibility have been viewed, but the disabled community prefers a social model approach focused on problem‐solving rather than assigning pity or blame.
- Definitions and categorizations of disability are many, and can depend on the context.
- Language for discussing disability should use neutral, straightforward terms, follow each community's self‐expressed preferences, and adapt to evolving norms, while respecting variations in individual preferences.
- Rather than memorizing “correct” terms, the focus should be consideration of individuals and the broader implications of what is said.
- Individuals involved in admissions or hiring are obligated to be aware of the ADA requirements, such as providing reasonable accommodations and avoiding inappropriate interview questions.
- There are many resources available for how to discuss accommodations with your students or staff, how to plan accessible meetings and events, and how best to advertise a disability‐welcoming environment. Some are linked here in the citations.

**FIGURE 4:**
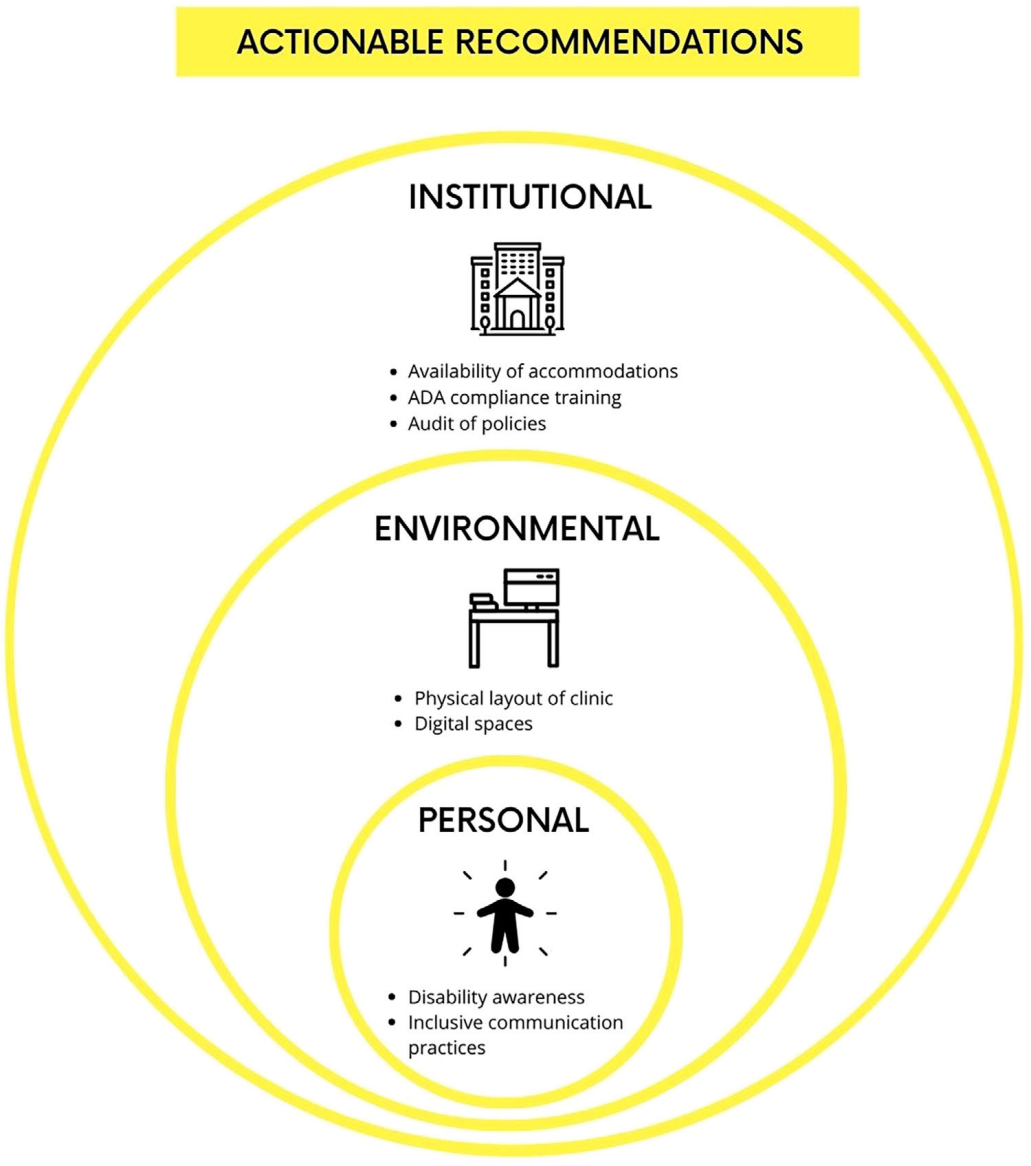
Actionable recommendations for disability and accessibility inclusion on an institutional, environmental, and personal level.

To make the actionable recommendations more targeted to medical physics practice environments, they can be categorized into institutional, environmental, and personal levels (Figure 4). At the institutional level, policies and procedures should explicitly outline the availability of accommodations for disabled employees, trainees, and patients. For instance, institutions should establish clear guidelines on how to request adaptive tools, such as speech‐to‐text software for documentation or ergonomic workstations for individuals with mobility impairments. Additionally, hiring practices should include training on ADA compliance for those involved in recruitment, ensuring fair and inclusive interviews. Institutions should also conduct regular audits of policies to identify and eliminate barriers that disproportionately affect disabled individuals, fostering a culture of accessibility and inclusion. At the environmental level, the physical layout of clinical practice spaces, such as imaging suites, hot labs, and treatment consoles, should be assessed and modified to ensure accessibility. This could include installing height‐adjustable workstations, ensuring sufficient space for wheelchair maneuverability, and implementing visual or auditory signals for individuals with sensory disabilities. In addition, the digital environment can be made more accessible by adding alternative text to images and transcription to videos, selecting color schemes and layouts carefully, and ensuring compatibility with assistive technology. On a personal level, clinical staff should actively engage in self‐education on disability awareness and inclusive communication practices. For example, supervisors can be trained to discuss accommodations empathetically and effectively, colleagues can be encouraged to adopt respectful language in day‐to‐day interactions, and everyone can be provided implicit bias training on a regular basis. These changes not only reduce barriers for disabled individuals but also create a more equitable and supportive environment for all members of the medical physics community.

## 2 CONCLUSION

The medical physics profession has many timepoints that act as a barrier to progression to those with disabilities. It is important to be aware of these points as well as the steps to reduce the difficulty and ensure fairness for all in the field. Building a more just and effective future for medical physics requires addressing systemic barriers, including those related to disability. As the pipeline of education, training, and professional development for medical physicists is increasingly being refined to promote standards of excellence, it is essential to consider how each step may be an obstacle–or opportunity–for disabled people to contribute.

## AUTHOR CONTRIBUTIONS

All authors were responsible for conceptualization, methodology, investigation, resources, and writing (review and editing). The first author was also responsible for writing (original draft). The second author was responsible for supervision, the fourth author was responsible for visualization (figure creation), and the senior author was responsible for publication support.

## CONFLICT OF INTEREST STATEMENT

The authors declare no conflicts of interest.
